# Current Insights and Progress in the Clinical Management of Head and Neck Cancer

**DOI:** 10.3390/cancers14246079

**Published:** 2022-12-10

**Authors:** Mariana Neves Amaral, Pedro Faísca, Hugo Alexandre Ferreira, Maria Manuela Gaspar, Catarina Pinto Reis

**Affiliations:** 1 Research Institute for Medicines (iMed.ULisboa), Faculty of Pharmacy, Universidade de Lisboa, 1649-003 Lisbon, Portugal; 2 Instituto de Biofísica e Engenharia Biomédica (IBEB), Faculdade de Ciências, Universidade de Lisboa, Campo Grande, 1749-016 Lisbon, Portugal; 3 Laboratório Veterinário, Faculdade de Medicina Veterinária—Universidade Lusófona de Humanidades e Tecnologias/DNAtech, 1749-024 Lisbon, Portugal

**Keywords:** head and neck cancer, head and neck squamous cell carcinoma, current treatments, novel therapies

## Abstract

**Simple Summary:**

Head and neck cancer (HNC) incidence has been steadily increasing since the 1990s. While the multimodal treatment approach for localized HNC is well established and renders a good treatment response, this is not the case for advanced or recurrent/metastatic HNC. Most patients present HNC at an advanced stage at the time of diagnosis, and the lack of effective treatment results in the death of half of patients diagnosed with advanced or recurrent/metastatic HNC. This review aims to present a current summary of the epidemiology, diagnosis, histopathology, current treatment and novel treatment approaches for HNC.

**Abstract:**

Head and neck cancer (HNC), also known as the cancer that can affect the structures between the dura mater and the pleura, is the 6th most common type of cancer. This heterogeneous group of malignancies is usually treated with a combination of surgery and radio- and chemotherapy, depending on if the disease is localized or at an advanced stage. However, most HNC patients are diagnosed at an advanced stage, resulting in the death of half of these patients. Thus, the prognosis of advanced or recurrent/metastatic HNC, especially HNC squamous cell carcinoma (HNSCC), is notably poorer than the prognosis of patients diagnosed with localized HNC. This review explores the epidemiology and etiologic factors of HNC, the histopathology of this heterogeneous cancer, and the diagnosis methods and treatment approaches currently available. Moreover, special interest is given to the novel therapies used to treat HNC subtypes with worse prognosis, exploring immunotherapies and targeted/multi-targeted drugs undergoing clinical trials, as well as light-based therapies (i.e., photodynamic and photothermal therapies).

## 1. Introduction

Head and neck cancer (HNC), the 6th most common type of cancer, also commonly known generating the malignant tumours between the dura mater and the pleura, affects structures such as the naso-, oro- and hypopharynx, larynx, nasal cavity, oral cavity, the floor of the mouth, the palate, tongue, tonsils, oesophagus, middle ear, paranasal sinuses, salivary glands, thyroid gland skin (melanoma), etc. [1,2,3,4,5,6]. Furthermore, the histopathology of HNC varies according to the structures affected, and thus HNC is also characterized as presenting molecular heterogeneity. Due to the low awareness of HNC, most cases are identified at advanced stages, resulting in death for half of all the patients diagnosed with this malignancy [7].

## 2. Epidemiology and Etiology

**Figure 1:**
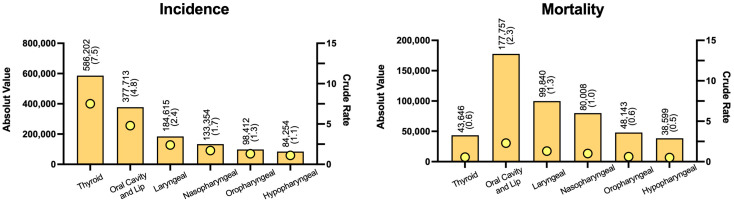
Incidence, mortality, and respective crude rates of the most prevalent subtypes of Head and Neck Cancer (HNC) worldwide in 2020, according to the Global Cancer Observatory (GCO), as determined by the International Agency for Research on Cancer (IARC).

As mentioned, HNC is a diverse family of cancers affecting this very broad anatomical region. However, as a whole, HNC is one of the mostly occurring frequent malignancies in the world [8,9]. Yearly, HNC accounts for more than 830,000 cases, which translates into 8% of all cancer diagnosis, and at least 430,000 deaths worldwide [3,4,5,10]. These numbers are expected to rise, as the HNC incidence has been steadily increasing since the 1990s in many countries, and this seems to be the trend worldwide [11,12,13,14]. The incidence and mortality of each malignancy comprised in the HNC group varies according to the exposure to risk factors, demographics and geography, and the epidemiologic data regarding the most prevalent subtypes of HNC in 2020 is presented in Figure 1 [8]. Although less prevalent, lymphomas are the second most common group of malignancies affecting the head and neck, next to malignancies of epithelial origin, accounting for 15% of all HNC diagnosis [15,16,17,18]. Most head and neck lymphomas affect the anatomical structures of the Waldeyer’s ring (base of the tongue, soft palate, pharynx and the tonsils) [17,18,19]. Sarcomas are a rare type of cancer arising from bone and soft tissues. They represent 1% of all cancer diagnoses, and only 10% of sarcomas occur in the head and neck, thus constituting a rare subtype of HNC [20,21,22]. Less prevalent subtypes of HNC also include major salivary gland cancer, accounting for 1–5% of cases, and sinonasal malignancies of various origins (i.e., carcinomas, lymphomas and sarcomas), representing less than 5% of all HNC diagnosis [23,24,25,26]. Moreover, the incidence of HNC is higher in men than in women and seems to increase with age [27]. The gender disparities in HNC incidence has been connected to the higher exposure to risk factors, such as alcohol and/or tobacco consumption, by men when compared to women [28,29]. 

The etiological factors related to HNC are summarized in Table 1 and can be classified as non-infectious or infectious [1,5]. There are different non-infectious risk factors for the development of HNC, the most important being tobacco and alcohol use. Although they contribute to the risk of HNC when consumed separately, their combination results in a synergistic increase in the risk of developing HNC [3,5,30,31]. Approximately 90% of all HNC patients have a history of tobacco consumption (i.e., smoked, snuffed or chewed) [27]. Different forms of tobacco consumption increase the risk of development of HNC differently, i.e., five- to ten-fold for smoked tobacco and four-fold for smokeless tobacco (if consumed for ≥10 years) [27,32]. Alcohol is another non-infectious etiological factor for the development of HNC [31]. When combined, alcohol and tobacco consumption can increase the risk of HNC by more than 40-fold [33]. This increase in risk, related to the combined use of alcohol and tobacco, is due to the fact that alcohol acts as a solvent to the tobacco substances, enhancing mucosal exposure to carcinogens [33]. There are some subtypes of HNC more prevalent amongst alcohol and/or tobacco users, such as laryngeal and oropharyngeal cancers, and some subtypes of head and neck squamous cell carcinoma (HNSCC) [34]. Moreover, some risk factors have been directly linked to certain subtypes of HNC, as is the case of salivary HNC and occupational exposure to radiation [35,36]. It is also important to note that the radiation exposure related to diagnostic testing is thought to be a contributing factor to the development of salivary cancer [35]. Poor oral and dental hygiene also represent another type of non-infectious risk factor for the development of HNC as it will lead to oral infections and to the presence of polymicrobial supragingival plaque, promoting cancer development [33,37]. Solar exposure, especially exposure to UV radiation, is also a known risk factor for the development of HNC, namely HNC affecting the skin [38]. There are also etiologic factors that are region-specific, as is the case of betel nut chewing, an etiologic factor highly specific to Asian countries [39,40].

The main infectious risk factor associated with the development of HNC are infections by oncogenic forms of HPV, particularly HPV16 (>90% of HPV-positive HNSCC cases) and HPV18 [41,42,43]. HPV infection seems to especially increase the risk of developing HNSCC [42]. In the past, the majority of HNSCC diagnoses were related to non-infectious etiological factors. However, recently, there has been a change in the paradigm, with a rise in HPV-related HNSCC. Indeed, HPV seems to be the main etiological factor driving carcinogenesis for most HNSCC cases, especially in young adults [44,45]. The anatomical structures presenting a higher incidence of HPV-related HNSCC include the oro- and hypopharynx [33,46,47]. Although HPV-related HNC patients tend to be younger, and smoke and drink less than non-HPV-related HNC patients, there are still HNC diagnoses simultaneously related to non-infectious and infectious etiological factors. In fact, drinkers and/ or smokers that are HPV-positive account for 10–30% of all HNSCC cases, and the combination of non-infectious and infectious risk factors seem to have an additive effect [41,48].

EBV, also an infectious risk factor for the development of HNC, has been strongly associated with nasopharyngeal cancer [49,50]. The first association between nasopharyngeal cancer and EBV was described in 1996, and EBV infection is strongly related to the undifferentiated subtype of this malignancy, with latent EBV being present in 95% of cases [51]. Many EBV infections occur during childhood, with the individual becoming a lifelong carrier of this virus as the virus becomes latent and survives in the pool of infected memory B cells [52]. Over time, EBV is able to revert from its latent state to a lytic state, acting as a tumour promoting-agent, and causing cells to transform and originate malignant tumours, such as nasopharyngeal carcinomas [49,50,52].

## 3. Clinical Presentation and Diagnosis

### 3.1. Diagnosis

HNC patients usually display symptoms related to trouble and/or pain in swallowing (dysphagia and odynophagia, respectively), hoarseness, otalgia, irregular mucosae, mucosae ulcers, oral and/or pharyngeal pain, weight loss, occurrence of unexplained neck mass, etc., usually presented to their primary care physician or dentists [3,53,54]. When justified, imaging techniques are generally employed prior to biopsies [3]. Due to the vast group of anatomical structures involved in this pathology, the diagnosis of HNC uses many techniques, traditionally nasopharyngolaryngoscopy, sinuses contrast-enhanced computed tomography (CT), head magnetic resonance imaging (MRI) and/or CT, panoramic dental X-ray, PET/CT, and chest imaging using different techniques [55,56]. If a biopsy is required, fine-needle aspiration is preferable for histological analysis. Nevertheless, complete nodal resection may be required [3].

Less invasive techniques for HNC diagnosis are being studied, such as serological and salivary biomarkers, as well as exhaled breath analysis and liquid biopsies [57,58,59]. The latter can be performed by the NA-NOSE, a nanoscale artificial nose developed by Haick and co-workers, that has already been shown to be able to distinguish between breathing of healthy cohorts and breast, lung, prostate and colon cancer [60], and more recently between healthy cohorts and HNC patients [56,61]. Some biomarkers have been shown to have diagnostic and prognosis-predictive value, including epidermal growth factor receptor (EGFR), HPV, EBV, phosphatase and tensin homolog (PTEN), p16, interleukin-8 (IL-8), B-cell lymphoma extra-large (Bcl-xL)/Bcl-2, and the upregulation of genes (i.e., EMS1, CCDN and FGFR1) [55,62]. Although the relevancy of some of these biomarkers for HNC prognosis has been confirmed (i.e., IL-8, HPV and p16), their detection in biological samples remains a challenge due to their low concentrations [55]. Another technique being studied extensively for treatment of HNC and its subtypes is liquid biopsies. These enable diagnostic and prognosis/predictive applications, making use of circulating tumour cells, circulating tumour DNA, micro-RNAs, extracellular vesicles, etc. [59]. Liquid biopsies have been suggested as a useful and less-invasive tool to track the malignancy and to obtain samples from less-accessible tumours. Moreover, they allow researchers to track tumour changes during the disease course, allowing them to monitor the treatment response, disease progression and the likelihood of disease relapse [63,64]. A major challenge in the screening and early detection of HNC is the lack of specific tumour markers that may be overcome using liquid biopsies. For instance, patients’ urine may be used to detect the presence of EBV-DNA, a biomarker for the diagnosis of nasopharyngeal cancer presenting a sensitivity of 96%, and thus constituting a promising non-invasive screening strategy for this subtype of HNC [64]. Besides their role in the treatment of nasopharyngeal cancer, liquid biopsies have also been explored to detect HNSCC, especially using blood (plasma or serum) and saliva, thus providing a vast list of biomarker candidates through the quantification of cell-free DNA (i.e., cfHPV DNA), cell-free RNA, circulating tumour cells and extracellular vesicles [65,66]. There has been cargo of extracellular vesicles associated with the onset and development of certain subtypes of HNC, as is the case of HNSCC and associated proteins (CD9, EpCAM, HSP90, FGFR2, CAV1 and gp96), micro RNAs (miR-486–5p, miR-486–3p, miR-10b-5p, miR-142–3p, miR-186–5p, miR-195–5p, miR-374b-5p, miR-574–3p and miR-21), long non-coding RNA (MALAT1, Linc-ROR and lncRNA00152) and circular RNA (circRNA_100290); nasopharyngeal cancer and associated proteins (LMP1) and micro RNAs (miR-BART7-3p, hsa-miR-24–3p, hsa-miR-891ª, hsa-miR-106a-5p, hsa-miR-20a-5p and hsa-miR-1908); and, thyroid carcinoma and associated proteins (HSP27, HSP60, HSP90, SRC, TLN1, ITGB2 and CAPNS1) and circular RNA (hsacirc_007293, hsacirc_031752 and hsacirc_020135) [67].

### 3.2. An Overview of the Pathophysiology and Histology of HNC

Overall, HNC staging follows the TNM system, taking into consideration the tumour characteristics (T), the presence of lymph nodes metastasis (N) and distant metastasis (M), and also HPV and EBV status for the staging of naso- and oropharynx HNC [68,69]. Over the years, the American Joint Committee on Cancer (AJCC)’s TNM system has been evolving to increase the system’s predictive value, and currently staging of HNC follows the 8th Edition of the AJCC, as described in Table 2.

Histopathologic analysis is very important for an adequate diagnosis and treatment of HNC, as well as the identification of key predictive or prognostic markers, as specific tumours present typical histological patterns [71,72]. For instance, HPV (i.e., HPV+ oropharynx HNSCC has a more favorable prognosis compared to HPV), EBV (i.e., EBV+ has a worse prognosis than EBV-), protein p16 (i.e., p16+ HNSCC present poorer differentiation but better prognosis), and EGFR (i.e., overexpression is correlated with increased recurrence and decreased survival) are some of the most relevant predictive and/or prognostic markers in HNSCC [73,74]. Moreover, it is important to score the tumour according to one of four grades (grade 1, 2, 3 or 4). This grading system varies per the HNC subtype (i.e., SCC, carcinoma, adenocarcinoma, sarcomas, and lymphomas). For instance, according to Anneroth’s (1987) and Bryne’s (1992) grading systems, to grade HNSCC the pathologist must take into account the degree of keratinization, the presence of nuclear polymorphisms, the number of mitoses in a high-power field, the pattern of growth and/or invasion of the surrounding tissues and the presence of lymphoplasmacytic infiltration [75,76]. In contrast, larynx and other HNC neuroendocrine carcinomas are graded according to their tumour subtype (i.e., grade 1—well-differentiated neuroendocrine carcinoma; grade 2—moderately differentiated neuroendocrine carcinoma; grade 3—poorly differentiated small- or large-cell neuroendocrine carcinomas) [77]. Similar to neuroendocrine HNCs, sarcomas of the head and neck are also graded according to their differentiation degree [78]. There is a large variety of histopathological subtypes of HNC, summarized in Table 3, as well as variety in their histopathological presentation. Due to the large variety of malignancies affecting the head and neck anatomical structures, one of the most important features to consider for histopathological diagnosis and subtyping is to the anatomical structure where the mass is localized.

### 3.3. Current Treatment Approaches

**Figure 2:**
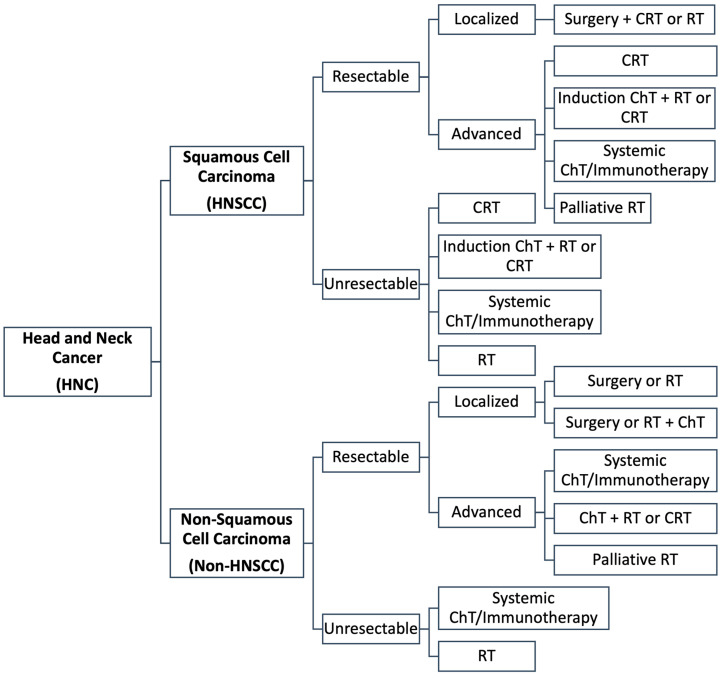
Generalized guidelines for the treatment of HNC (CRT—Chemoradiotherapy, RT—Radiotherapy and ChT—Chemotherapy).

Like other malignancies, the treatment of HNC differs according to subtype, primary site and disease stage (i.e., localized or locally advanced disease), and is described in the NCCN Clinical Practice Guidelines in Oncology [4,81,82,83]. A generalized representation of the different treatment approaches for HNC is presented in Figure 2. Regarding localized HNC (TNM stage I-II), treatment is very similar regardless of the anatomical structure affected (i.e., lip, oral cavity, oropharynx, larynx, hypopharynx and paranasal sinuses) or malignancy subtype (i.e., epithelial, HNSCC, lymphoma or sarcoma), focusing on treating the primary tumour site by complete surgical resection or with radiotherapy (if the patient is unfit for surgery) [3,84]. Patients that present with localized HNC of epithelial nature usually have improved long-term survival prospects (70–90% of patients with early-stage disease) after surgery or radiotherapy. In all cases, the risk of occult neck metastasis is evaluated, with selective neck dissection, elective neck dissection with more extensive removal of lymph nodes or prophylactic neck radiotherapy be potentially offered [3]. Regarding the specific case of soft tissue sarcomas of the head and neck, neck metastasis is rare (3% of cases), and thus neck dissection is only indicated when palpable lymph nodes are identified [78]. In the case of high-grade soft tissue sarcomas, chemotherapy is also offered in combination with surgery and radiotherapy [78,85]. Regarding lymphomas of the head and neck, treatment differs from the regimens of other solid HNCs and mainly depends on the malignancy being low- or high-grade. While localized low-grade lymphomas are treated solely with radiotherapy, localized high-grade lymphomas are treated with chemotherapy followed by radiotherapy [86]. The majority of extra-nodal lymphomas of the head and neck are treated with rituximab and CHOP, a multiagent chemotherapy regimen combining cyclophosphamide, doxorubicin or epirubicin, bleomycin and vincristine, or its variants (i.e., R-CHOP) [86,87]. In the case of very aggressive head and neck lymphomas, such as Burkitt’s lymphoma and lymphoblastic lymphoma, chemotherapy must be combined with central nervous system (CNS) prophylaxis due to high rates of CNS involvement, and in some cases, patients may be eligible for stem cell transplants. Hodgkin’s lymphoma is standardly treated with a combination of radiotherapy and multiagent chemotherapy, combining adriamycin, bleomycin, vinblastine and dacarbazine [87].

Patients with locally advanced disease (TNM stage III, IVa and IVb) are treated with a multimodal approach, including not only localized treatments (i.e., surgical resection and radiotherapy), but also systemic treatments (i.e., chemotherapy) [84,88]. A multidisciplinary approach for HNC treatment focuses on maximizing survival while preserving function and structures, with a team of surgeons, oncologists and radiotherapists being responsible for tumour control [53,89,90]. The main task of this team is to promote specialized decision making regarding diagnostic techniques, treatment options and treatment recommendations for each patient, establishing an adequate quality of life [90].

Regarding advanced HNSCC, the most commonly used chemotherapy agents are platinum-based, and can be used in combination with 5-fluorouracil (5-FU) to treat HNC. However, no platinum-based chemotherapy agent was shown to be significantly superior [88,91,92]. Moreover, in the case of advanced HNSCC, treatment with chemoradiation (i.e., chemotherapy as radiosensitizers before subsequent or concurrent radiotherapy) is very frequent [88,91]. EGFR is commonly overexpressed in HNSCC and has been associated with a poorer prognosis. Thus, cetuximab is routinely used in advanced HNSCC treatment and has been proven useful in platinum-resistant HNSCC treatment [92]. In fact, the addition of cetuximab to platinum-5-FU chemotherapy has been the only improvement in the clinical management of metastatic HNSCC, leading to increased survival rates [91]. To improve quality of life and provide symptomatic relief for these groups of patients, palliative radiotherapy may be provided to treat distant metastasis [92]. Still, regarding advanced HNSCC, HPV status does not change the treatment regimen, with HPV+ positive patients being treated similarly to their HPV- counterparts [92]. Some HPV+ positive HNC subtypes may have higher response rates to therapies, improved overall survival, disease-free survival and locoregional disease control when compared to their HPV- counterparts [93]. One example of this is oropharyngeal cancer, in which patients diagnosed with HPV+ oropharyngeal cancer have higher response rates to chemoradiation and increased survival [41]. This increased sensitivity to chemo- and radiotherapy has been attributed to the presence of unmutated p53 and higher sensitivity to cytotoxic agents and DNA damage, both of which induce apoptosis [41,93].

To maintain the patient’s quality of life, a multidisciplinary care team must be put in place, encompassing not only physicians specializing in surgery, oncology and radiotherapy, but also dentists, nutritionists, speech therapists, and occupational- and physiotherapy, audiometry and psychosocial services [3].

## 4. Therapeutic Advances in HNC

HNC continues to face major treatment challenges due to a variety of reasons, one of them being the high diversity of cancers englobed by this group of malignancies. They vary not only in the anatomical regions affected but also in histologic subtype, and also in HNC presenting early development of drug-resistance pathways, leading to treatment failure. Thus, although there are effective treatments available for localized HNC, with favorable response rates, the prognosis of advanced HNC, especially HNSCC, that relies in the response to systemic therapies (i.e., chemotherapy) is uncertain, mainly due to poor treatment response rates [94,95]. Besides low efficacy, the current treatment modalities (i.e., chemo- and radiotherapy) also present high toxicity, leading to adverse reactions to treatment and even premature treatment cessation. These challenges result in up to 65% of patients diagnosed with advanced HNSCC presenting a high risk of disease reoccurrence, and low survival rates; less than 50% of patients reach the 5-year survival mark [3].

The rationale behind immunotherapy is to induce or improve the anti-tumour response, as well as avoid immune evasion. The fact that HNSCC and other advanced HNC present an extremely immunosuppressive tumour microenvironment, high tumour mutation burden and high expression of immune checkpoint inhibitors (programmed cell death protein 1, PD-1; and cytotoxic T-lymphocyte-associated protein 4, CTLA4) makes them very attractive candidates for cancer immunotherapy [96,97]. Cetuximab was approved as a targeted therapy for recurrent/metastatic HNSCC after the EXTREME study, targeting EGFR, but it has shown limited efficacy in the clinical setting [81,94,98]. Soon after, in 2017, after the CheckMate 141 study, and in 2019, after the KEYNOTE-048 study, respectively, nivolumab was approved as a second-line treatment for platinum-resistant recurrent/metastatic HNSCC and pembrolizumab was approved as mono- and combination therapy for localized and recurrent/metastatic HNSCC, and both were checkpoint inhibitors targeting PD-1 [94,97,98,99,100]. Immunotherapy approaches using PD-L1 and PD-1 inhibitors (i.e., pembrolizumab and nivolumab) revolutionized the treatment of many solid malignancies, with durable treatment responses [101]. Immune checkpoint inhibitors seem to be promising therapeutic approaches for recurrent/metastatic HNSCC, as 85% of these tumours are positive for PD-L1 [97]. However, similar to cetuximab, these immune checkpoint inhibitors showed low efficacy in the clinical setting. Patients undergoing treatments with these drugs present median overall survivals of 7.7 months, for nivolumab, and 14.7–14.9 months, for pembrolizumab [94,98]. Nevertheless, patient outcomes were improved by using immune checkpoint inhibitors when compared with the standard therapies. The CheckMate 141 trial reported a doubling of the 1-year overall survival of patients that received nivolumab (36%) compared with conventional chemotherapy (17%), and a nearly tripling of the 2-year overall survival rate (17% versus 6%) [97,101,102].

Patients receiving nivolumab also reported fewer adverse effects of grade 3 and 4 (13.1%), indicating less toxicity than what is seen with conventional chemotherapy regimens (35.1%) [102,103]. The KEYNOTE-048 trial, that resulted in pembrolizumab’s approval for the treatment of recurrent/metastatic HNSCC, also reported, very importantly, that patients treated with this immune checkpoint inhibitor presented not only improved overall response to treatment and overall survival, but longer-lasting responses. A total of 85% of tumour responses to treatment lasted at least 6 months, and 71% of responses lasted a year or more [102]. Information drawn from clinical trials using immune checkpoint inhibitors to treat other metastatic malignancies (i.e., melanoma) indicate that some patients continue to benefit from these drugs long after treatment discontinuation, with tumours presenting long-term responses to treatment [104]. Currently, the FDA recommends immune checkpoint inhibitors for the treatment of recurrent/metastatic HNSCCs that are inoperable and are not candidates for radiotherapy, as follows: pembrolizumab plus cisplatin or 5-FU, if previous platinum chemotherapy resulted in a disease-free interval longer than 6 months and tumour is PD-L1 negative; pembrolizumab or pembrolizumab plus cisplatin or 5-FU, if previous platinum chemotherapy resulted in a disease-free interval longer than 6 months and tumour is PD-L1 positive; and nivolumab or pembrolizumab, if previous platinum chemotherapy resulted in a disease-free interval of 6 months or lower [97]. Now, there is the urgent need to identify which patients benefit from immunotherapeutic strategies such as immune checkpoint inhibitors, and also the optimal time points for these therapies (i.e., neoadjuvant, locally advanced disease, metastatic disease) [105]. This is still an ongoing challenge for researchers, but based on what is known regarding tumour response to immune checkpoint inhibitors by other cancers, tumour mutation burden (i.e., lung cancer) and microsatellite instability (i.e., colon cancer) seem to be the most useful markers besides PD-1/PD-L1 score [106,107].

Another immunotherapeutic approach under study to treat HNC are the therapeutic vaccines for HPV-related HNC, such as HPV+ oropharyngeal cancer. HPV16 is the only associated viral cancer-causing agent in this subtype of HNC, and thus some therapeutic vaccines have been developed for this malignancy. Different therapeutic vaccine types are undergoing clinical trials for their’ application in HPV+ oropharyngeal HNC, e.g., peptide-based vaccine (registered in ClinicalTrials.gov under the identifier NCT00257738), DNA vaccine (registered in ClinicalTrials.gov under the identifier NCT01493154) and live-attenuated virus vaccine (registered in ClinicalTrials.gov under the identifier NCT01598792) [4,8,97,99,100,108,109]. Moreover, other therapeutic vaccines are undergoing clinical trials for the treatment of HNC, targeting HPV, EBV, MUC1, p53 and others [108]. In the case of EBV+ nasopharyngeal carcinoma, adoptive and active immunotherapeutic approaches have also been studied. The first is based on activating EBV-specific T cells ex vivo, which are passively transferred to patients after expansion. Active immunotherapeutic approaches are based upon loading autologous immune cells, with EBV antigens and transferring these cells to patients [8,99,100,109,110].

Besides immunotherapy, many biological-targeted therapies are under study for HNC, focusing on targeting growth factors and growth factor receptors, signal transduction, cell cycle targets, prostaglandin production, protein degradation, hypoxia and angiogenesis, etc. [91]. Examples of drugs and targets of interest of biological-targeted agents for HNC currently undergoing clinical trials are presented in Table 4, either as a single or combined therapy. Moreover other drugs have undergone clinical trials for HNC in the past, including PI3K inhibitors (i.e., metformin, parsaclisib, copanlisib, sonolisib, piliralisib); mTOR inhibitors (i.e., sirolimus/rapamycin, everolimus, CC-115, temsirolimus, ridaforolimus); AKT inhibitors (i.e., MK-2206, perifosine, temsirolimus); JAK/STAT (i.e., ruxolitinib, danvatirsen, TTI-101, itacitinib); dual PI3K/mTOR inhibitors (i.e., dactolisib, bimiralisib, PF-04691502, gedatolisib, voxtalisib, SF1126); VEGFR inhibitors (i.e., vandetanib, sunitinib, pazopanib, axitinib, nintedanib, cediranib, semaxinib); MET inhibitors (i.e., capmatinib, ficlatuzumab, AMG337, golvatinib, foretinib, tivantinib, trametinib, mirdametinib); EGFR inhibitors (i.e., panitumumab, afatanib, gefinitib, zalutumumab, tarloxotinib, nimotuzumab, DBPR112, iressa); Smoothened inhibitor (i.e., saridegib, vismodegib); Bcl-Arb inhibitor (i.e., imatinib, ponatinib, dasatinib, nilotinib); CDK4/6 inhibitors (i.e., abemaciclib, flavopiridol, riviciclib, palbociclib, ribociclib); NADPH inhibitor (i.e., setanaxib); and SUMOylation inhibitors (i.e., subasumstat), etc. [81,111,112,113,114,115,116,117,118,119,120,121,122,123,124,125,126,127,128,129,130,131,132,133,134].

Alternative treatment approaches are also being explored for the treatment of HNC. This is the case with light-based therapies, such as photodynamic- and photothermal therapy. Photodynamic therapy (PDT) is used to treat malignant and non-malignant diseases by producing reactive oxygen species through the light-mediated excitation of photosensitizer drugs [135]. PDT mainly presents clinical applications as second-line treatment options for second primary or recurrent superficial HNC, especially of the oral cavity, pharynx and larynx [136,137]. In both the oral cavity and the larynx, PDT is of special interest in the case of diffuse but superficial field cancerization [137,138]. For well-defined disease in the oral cavity, PDT’s efficacy is competitive with surgery, while in the larynx PDT can be used as a primary treatment when surgical excision proves difficult [137]. Photosensitizer drugs have been approved for PDT for curative treatment of esophageal cancer (Photofrin^®^) and early-stage HNSCC (Foscan^®^), and palliative treatment of advanced HNSCC (Foscan^®^) [137,138,139]. In early-stage HNSCC (T1 and T2 tumours), PDT response rates and cure rates are similar to those observed with conventional therapy, 79–91% and 71–89%, respectively. When conventional therapy is compared to PDT in advanced and incurable HNSCC, studies have reported a longer survival time with improved quality of life when PDT is preferred. [138,140] However, despite its benefits, PDT presents several drawbacks for HNC management. One of these is a high rate of recurrence in patients after PDT treatment [138]. This is most likely due to the limitation of light penetration in tissues (less than 5 mm), as inefficiently reaches the as a consequence tumour [137,138]. Another drawback associated with PDT is the severe acute pain at the treatment site reported by patients and pronounced swelling, attributed to high inflammatory response post-PDT of the tumour and tissues surrounding it [138].

Photothermal therapy (PTT) is another type of light-based therapy that entails the use of a light source to irradiate and increase the temperature of a superficial tissue (i.e., tumour), leading to thermal ablation through the excitation of endogenous chromophores [141,142,143,144,145,146,147,148]. Contrary to PDT, with photosensitizers approved by regulatory agencies like EMA and FDA to be used clinically, PTT is yet to be approved for clinical applications [147]. To increase PTT’s specificity and efficacy, photothermal nanoparticles (i.e., gold nanoparticles, AuNPs) able to convert light into heat can be used [142,143,144,145,146,149]. Different applications of AuNPs-based PTT for HNC have been studied in vitro [144,150,151] and in vivo [144,152,153], with some systems undergoing clinical trials [151,154]. Our group has previously developed AuNPs with high specificity for a rare subtype of thyroid cancer (part of HNC), anaplastic thyroid carcinoma, for AuNPs-based PTT of this aggressive malignancy with promising results, both in vitro and in vivo [144]. Moreover, other forms of AuNPs-based PTT are under clinical evaluation. One example is Aurolase^®^ (Nanospectra), a PEGylated gold nanosphere that underwent a pilot study clinical trial, concluded in 2014, for PTT of refractory and/or recurrent HNC (registered in ClinicalTrials.gov under the identifier NCT00848042) [151,154]. Besides monotherapy with PTT, applications of AuNPs-based PTT for HNC also include combining PTT with other therapies (i.e., radio-, chemo- and immunotherapy) and theranostic approaches, combining imaging and therapy [155,156].

## 5. Conclusions

The incidence of HNC has been steadily increasing since the 1990s, and thus this vast group of malignancies has become an important issue. Although the treatment for localized/early-stage HNC is well established, most patients present advanced and/or recurrent/metastatic disease when diagnosed, resulting in survival rates below 50%. Although efforts have been made to improve treatment for advanced HNC, little progress has been achieved as the efficacy of the newly approved therapies did not translate to the clinical setting. Currently, the focus on clinical trials for advanced and/or recurrent/metastatic HNC has shifted to targeted/multi-targeted therapies, immunotherapy and light-based therapeutic approaches (i.e., PDT and PTT). These strategies aim to improve the quality of life of these patients as well as the management of this challenging group of malignancies.

## Figures and Tables

**Table 1:** Etiologic factors of HNC.

**Non-infectious Risk Factors**	Substance Use	Tobacco
		Betel chewing
		Alcohol
	Behavioural	Poor oral and dental hygiene
		Solar exposure
	Dietary Deficiencies	Inadequate nutrition
		Vitamin A
		Iron (in Plummer–Vinson Syndrome)
	Occupational Hazards	Asbestos
		Radium
		Mustard Gas
		Nickel
		Radiation
		Leather tanning by-products
		Woodworking by-products
		Chromium
**Infectious Risk Factors**		HPV
		EBV

HPV—Human Papillomavirus; EBV—Epstein–Barr virus.

**Table 2:** TNM staging system of Head and Neck cancer (HNC) according to the 8th Edition of the American Joint Committee of Cancer (AJCC) Staging Manual (2017) [70].

Anatomical Location		T		N	M	Stage
**Unknown**		T0 (location unknown)		N1 (≤3 cm ipsilateral nodal metastasis and ENE-)	M0	III
				N2 (3–6 cm ipsilateral nodal metastasis and ENE-)	M0	IVA
				N3 (≥6 cm ipsilateral nodal metastasis and ENE-, or any ENE+ nodal metastasis)	M0	IVB
				Any N	M1	IVC
**Lip and Oral Cavity**		T1 (tumour ≤ 2 cm, DOI ≤ 5 mm)		N0 (no regional nodal metastasis)	M0	I
		T2 (tumour ≤ 2 cm, DOI 5–10 mm)				II
		T3 (tumour > 4 cm or DOI >10 mm)				III
		T1,2,3		N1 (≤3 cm ipsilateral nodal metastasis and ENE-)		
		T4a (moderate local disease)		N0,1		IVA
		T1,2,3,4a		N2 (3–6 cm ipsilateral nodal metastasis and ENE-, or multiple ≤6 cm ipsilateral nodal metastasis and ENE-, or ≤6 cm bilateral or contralateral nodal metastasis)		
		Any T		N3 (≥6 cm ipsilateral nodal metastasis and ENE-, or any ENE+ nodal metastasis)		IVB
		T4b (very advanced local disease)		Any N		
		Any T			M1	IVC
**Major Salivary Glands**		Tis (carcinoma in situ)		N0 (no regional nodal metastasis)	M0	0
		T1 (tumour ≤ 2 cm, w/o extraparenchymal extension)				I
		T2 (tumour 2–4 cm, w/o extraparenchymal extension)				II
		T3 (tumour ≥ 4 cm, and/or with extraparenchymal extension)				III
		T0,1,2,3		N1 (≤3 cm ipsilateral nodal metastasis and ENE-)		
		T4a (moderate local disease)		N0,1		IVA
		T0, 1, 2, 3, 4a		N2 (3–6 cm ipsilateral nodal metastasis and ENE-, or multiple ≤ 6 cm ipsilateral nodal metastasis and ENE-, or ≤ 6 cm bilateral or contralateral nodal metastasis)		
		Any T		N3 (≥6 cm ipsilateral nodal metastasis and ENE-, or any ENE+ nodal metastasis)		IVB
		T4b (very advanced local disease)		Any N		
		Any T			M1	IVC
**Nasopharynx**		Tis		N0 (no regional nodal metastasis)	M0	0
		T1 (tumour limited to the naso-, or with locoregional invasion of the oropharynx and nasal cavity)				I
		T1,0 (tumour not identified, EBV+ cervical nodes)		N1 (≤6 cm unilateral cervical lymph nodes metastasis, or uni-/bilateral retropharyngeal lymph nodes metastasis, above the cricoidal cartilage caudal border)		II
		T2 (tumour invading parapharyngeal space and/or adjacent soft tissue)		N0		
				N1		
		T1, 0		N2 (≤6 cm bilateral retropharyngeal lymph nodes metastasis above the cricoidal cartilage caudal border)		III
		T2				
		T3 (tumour invading surrounding bone structures)		N0		
				N1		
				N2		
		T4 (tumour with extensive soft tissue invasion and/or with intracranial extension)		N0		IVA
				N1		
				N2		
		Any T		N3 (>6 cm uni-/bilateral retropharyngeal lymph nodes metastasis and/or below the cricoidal cartilage caudal border)		
				Any N	M1	IVB
**HPV+ Oropharynx**		T0,1,2 (tumour not identified; tumour ≤ 2 cm or 2–4 cm)		N0,1 (no regional nodal metastasis; one or more ≤6 cm ipsilateral nodal metastasis)	M0	I
				N2 (≤6 cm contralateral or bilateral nodal metastasis)		II
		T3 (tumour < 4 cm or with invasion of epiglottis lingual surface)		N0,1,2		
		T0,1,2,3,4		N3 (>6 cm nodal metastasis)		III
		T4 (moderate locoregional invasion)		N0,1,2,3		
		Any T		Any N	M1	IV
**HPV- Oropharynx and Hypopharynx**		Tis (carcinoma in situ)		N0 (no regional nodal metastasis)	M0	0
		T1 (tumour ≤ 2 cm)				I
		T2 (tumour 2–4 cm)				II
		T3 (tumour < 4 cm or with invasion of epiglottis lingual surface)				III
		T1,2,3		N1 (≤3 cm ipsilateral nodal metastasis and ENE-)		
		T4a (tumour with moderate locoregional invasion)		N0,1		IVA
		T1,2,3,4a		N2 (3–6 cm ipsilateral nodal metastasis and ENE-, or multiple ≤ 6 cm ipsilateral nodal metastasis and ENE-)		
		Any T		N3 (≥6 cm ipsilateral nodal metastasis and ENE-, or any ENE+ nodal metastasis)		IVB
		T4b (very advanced locoregional invasion)		Any N		
		Any T			M1	IVC
**Nasal Cavity and Paranasal Sinuses**		Tis (carcinoma in situ)		N0 (no regional nodal metastasis)	M0	0
		T1 (limited to one subsite, with or without bone invasion)				I
		T2 (two subsites or invading adjacent subsites, with or without bone invasion)				II
		T3 (locoregional invasion of orbital medial wall or floor, maxillary sinus, palate, and cribriform plate)				III
		T1,2,3		N1 (≤3 cm ipsilateral nodal metastasis and ENE-)		
		T4a (moderate locoregional invasion)		N0,1		IVA
		T1,2,3,4a		N2 (3–6 cm ipsilateral nodal metastasis and ENE-, or multiple ≤ 6 cm ipsilateral nodal metastasis and ENE-, or ≤ 6 cm bilateral or contralateral nodal metastasis)		
		Any T		N3 (≥6 cm ipsilateral nodal metastasis and ENE-, or any ENE+ nodal metastasis)		IVB
		T4b (very advanced locoregional invasion)		Any N		
		Any T			M1	IVC
**Larynx** **(T classification depends on tumour location: supraglottis, glottis, subglottis)**		Tis (carcinoma in situ)		N0 (no regional nodal metastasis)	M0	0
		T1				I
		T2				II
		T3				III
		T1,2,3		N1 (≤3 cm ipsilateral nodal metastasis and ENE-)		
		T4a		N0,1		IVA
		T1,2,3,4a		N2 (3–6 cm ipsilateral nodal metastasis and ENE-, or multiple ≤ 6 cm ipsilateral nodal metastasis and ENE-, or ≤ 6 cm bilateral or contralateral nodal metastasis)		
		Any T		N3 (≥6 cm ipsilateral nodal metastasis and ENE-, or any ENE+ nodal metastasis)		IVB
		T4b		Any N		
		Any T			M1	IVC
**Cutaneous HNSCC**		Tis (carcinoma in situ)		N0 (no regional nodal metastasis)	M0	0
		T1 (tumour ≤ 2 cm)				I
		T2 (tumour 2–4 cm)				II
		T3 (tumour ≥ 4 cm, or minor bone invasion, or perineural invasion, or deep soft tissue invasion)				III
		T1		N1 (≤3 cm ipsilateral nodal metastasis and ENE-)		
		T2				
		T3				
		T1		N2 (3–6 cm ipsilateral nodal metastasis and ENE-, or multiple ≤ 6 cm ipsilateral nodal metastasis and ENE-, or ≤6 cm bilateral or contralateral nodal metastasis)		IV
		T2				
		T3				
		Any T		N3 (≥6 cm ipsilateral nodal metastasis and ENE-, or any ENE+ nodal metastasis)		
		T4 (major cortical bone/marrow invasion, skull base invasion or skull base foramen invasion)		Any N		
		Any T			M1	
**Thyroid Cancer**	**Differentiated**	<55 years old	Any T	Any N	M0	I
					M1	II
		≥55 years old	T1 (tumour ≤ 2 cm, confined to the thyroid)	N0/NX (no regional nodal metastasis; regional lymph nodes cannot be assessed)	M0	I
				N1 (regional nodal metastasis)		II
			T2 (tumour 2–4 cm, confined to the thyroid)	N0/NX		I
				N1		II
			T3 (tumour ≥ 4 cm, confined to the thyroid or locoregional invasion of surrounding muscles)	Any N		
			T4a (gross extrathyroidal invasion of subcutaneous soft tissues, larynx, trachea, oesophagus)			III
			T4b (gross extrathyroidal invasion of prevertebral fascia, or encasing of the carotid artery or mediastinal vessels of any size)			IVA
			Any T		M1	IVB
	**Anaplastic** **(T and N w/ same definition as for differentiated thyroid carcinoma)**	T1,2,3a		N0/X	M0	IVA
				N1		IVB
		T3b		Any N		
		T4				
		Any T			M1	IVC
	**Medullary** **(T and N with same definition as for differentiated thyroid carcinoma)**	T1		N0	M0	I
		T2				II
		T3				
		T1,2,3		N1a		III
		T4a		Any N		IVA
		T1,2,3		N1b		
		T4b		Any N		IVB
		Any T			M1	IVC

T—tumour; N—node; M—metastasis; ENE—Extranodal Extension; DOI—Depth of Tumour Invasion; EBV—Epstein–Barr virus; HPV—Human Papillomavirus; HNSCC—Head and Neck Squamous Cell Carcinoma; M0—no distant metastasis; M1—the presence of distant metastasis.

**Table 3:** Histopathology of HNC and their main characteristics [79,80].

Histopathological Subtype			Main Characteristics
Epithelial	Conventional	Squamous Cell Carcinoma	Characteristics of squamous epithelium that penetrated the basement membrane. Different degrees of differentiation.
	Verrucous		Resembles common wart with a pushing border and blunt bulbous projections in a chronically inflamed stroma.
	Papillary		Narrow papillae with fibrovascular cores (rare variant). HPV-positive or -negative.
	Basaloid		Cribriform nests of small cells with high nuclear to cytoplasm ratio (aggressive variant). Oropharyngeal forms are likely to be HPV+.
	Spindle cell		Polypoid mass with areas of conventional SCC (rare variant). Bone and cartilage may also be present.
	Lymphoepithelial		EBV+ undifferentiated carcinoma presents as a nest or single-cell population in the presence of lymphocytes.
	Intestinal-type	Sinonasal Adenocarcinoma	Resembles the aggressive form of colon adenocarcinoma.
	Non-intestinal-type		Resemble normal sero-mucinous glands, with solid growth pattern, necrosis and marked nuclear atypia.
	Sinonasal Undifferentiated	Carcinoma	Unknown etiology, presenting as sheets of mitotically active large cells with large nucleoli (aggressive variant). Sometimes presents p16 expression when negative for HPV.
	Nasopharyngeal		Keratinizing, non-keratinizing or non-keratinizing undifferentiated. The latter two are EBV+.
	Large Cell Neuroendocrine		Large cells with abundant cytoplasm and typical neuroendocrine immunophenotype.
	Small Cell Neuroendocrine		Resembles its lung namesake.
	Mucoepidermoid		Low-grade tumours usually present larger populations of mucous cells, while high-grade forms present epithelioid and intermediate cells. Translocation t(11;19) is a marker of favourable prognostic.
	Adenoid Cystic		Tumour of the major or minor salivary glands, presenting as tubules, cribriform or solid nests, with patterns of luminal or abluminal differentiation.
	Acinic Cell		Resembles the normal parotid gland, with absent ducts.
	Epithelial–Myoepithelial		Tumours with the highest degree of luminal and abluminal differentiation, with focal areas containing bi-layered ducts, with columnar epithelial and myoepithelial cells.
	Salivary Duct		Infiltrating cord, papillae, and large nests with necrosis. Usually expresses androgen receptor (AR), overexpresses HER2, and is negative for oestrogen (ER) and progesterone (PR) receptors.
	Polymorphous Low Grade	Adenocarcinoma	Tumour of the minor salivary glands similar to adenoid cystic carcinoma, but with polymorphous architecture, and no luminal or abluminal differentiation.
	Basal Cell		Prominent basement membrane and hyaline globules scattered within a nest architecture.
	Ceruminous Gland		Similar features to salivary gland carcinomas, presenting as an infiltrative cribriform nest, with luminal and/or abluminal cells.
	Olfactory Neuroblastoma		Uniform blue cells arranged in rosettes in neurofibrillary background. Poorly differentiated forms loose lobular architecture and present necrosis.
	Ewing’s Sarcoma		Small blue cells with t(11;22) translocation involving EWS and FLI-1 genes, seen in adolescents and young adults.
	Primitive Neuroectodermal Tumour		Similar to Ewing’s sarcoma but seen in any age group and presents neuroendocrine differentiation. Mutation in p53 signifies a poorer prognosis.
	Melanoma		Same presentation of melanoma at other sites.
	Carcinoma *ex* Pleomorphic Adenoma		A benign tumour mixed with adenocarcinoma, composed of epithelial and/or mesenchymal entities.
	Ameloblastoma		Odontogenic epithelium, resembling the enamel organ of developing tooth.
Mesenchymal	Osteosarcoma		Resembles osteosarcoma of other sites.
	Laryngeal	Chondrosarcoma	Lobulated nodules of cartilage, with increased cellularity with nuclear pleomorphism. Spindle cells may be present.
	Mesenchymal		Rare form with distinctive biphasic appearance, presenting as nodules of hyaline cartilage surrounded by sheets of small or spindle cells.
	Conventional	Chondromas	Pseudoencapsulated tumours with fibrous bands, with the cells growing in cords, sheets, or pseudo-glandular entities, in a mucinous matrix.
	Dedifferentiated		Like conventional chondromas, but lack mucinous matrix.
	Chondroid		Conventional chondrosarcoma with the addition of cartilage foci.
	Embryonal	Rhabdo-myosarcoma	A wide range of presentations, from undifferentiated cells to those resembling foetal muscle. Stains for skeletal muscle-specific markers.
	Botryoid		Polypoid masses, formed by hypocellular zones separated from the epithelium by a hypocellular layer of connective tissue.
	Alveolar		Small round cells separated by dense fibrous septae into nests, with dicohesive cells in the centre.
	Mesenchymal Extrarenal Rhabdoid Tumour		Rhabdoid cells and loss of nuclear INI1 expression.
	Alveolar Soft Parts	Sarcoma	Highly vascular tumours presenting as nests, formed by dicohesive cells, with haemorrhagic and necrotic areas.
	Synovial		t(X;18) (p11;q11) translocation.
	Follicular Dendritic Cell		Spindle cells present in fascicular, storiform or diffuse pattern, in the presence of lymphocytes.
Lymphoid	Conventional	Lymphoma	Can present as Hodgkin’s or non-Hodgkin’s lymphoma (NHL).
	Diffuse Large B-cell		Most common lymphoma of the head and neck, presenting as sheets/clusters of intermediate-to-large cells, with large necrotic areas.
	Plasmablastic		Rare variant of diffuse large B-cell lymphoma, presenting as large plasmocytic cells, proliferating diffusely, with abundant apoptotic bodies. They may also present necrotic regions.
	MALT		Small-to-medium lymphocytes in size with pale cytoplasm, or plasmacytic features. Translocation t(11;18) (q21;q21) is frequent.
	Extranodal NK-/-T-Cell of Nasal Type		Mixture of cells with different dimensions, irregular nuclei and amounts of pale cytoplasm, and chronic inflammatory infiltrates. The overlying mucosa usually presents as ulcerated but can range to hyperplastic. Commonly EBV+.
	Burkitt’s		Uniform mitotic lymphoid cells, with interspersed macrophages and phagocytized apoptotic tumour cells. Can be EBV+.
	Plasmacytoma		Sheets and nests of plasma cells, sometimes with plasmablastic features, and scattered multinucleated giant tumour cells.

SCC—Squamous Cell Carcinoma; HPV—Human Papilloma Virus; EBV—Epstein–Barr Virus; HNC—Head and Neck Cancer; AR—Androgen Receptor; ER—Oestrogen Receptor; PR—Progesterone Receptor; NHL—Non-Hodgkin’s Lymphoma; MALT—Mucosa-Associated Lymphoid Tissue; NK—Natural Killer T Cell.

**Table 4:** Examples of different targeted therapies in clinical trials for HNC and respective targets.

Target	Drug	Therapeutic Indication(s)	Phase	ClinicalTrials.Gov ID
PI3K	Buparlisib	R/M HNSCC	III	NCT04338399
	Alpelisib		II	NCT04997902
	Copanlisib		I	NCT03735628
	Eganelisib		I	NCT02637531
	Duvelisib	R/M and non-R/M HNSCC	II	NCT05057247
PI3K and mTOR	Gedatolisib	R/M or Advanced HNC	I	NCT03065062
AKT	Ipatasertib		I	NCT05172245 NCT05172258
			II	
p53	COTI-2	HNC	I	NCT02433626
	Advexin	R/M HNSCC	II	NCT03544723
EGFR	Erlotinib		II	NCT00076310 NCT01927744 NCT00954226
		HNSCC	II	
		HNC	I	
	Afatinib	R/M HNSCC	II	NCT02979977
		Resectable HNSCC	II	NCT05517330 NCT05516589
			II	
		R/M HNSCC	III	NCT01856478
	Dacomitinib		II	NCT04946968
	Lapatinib	Localized HNSCC	II	NCT01612351
		HNC	II	NCT01711658
PARP	Olaparib		I	NCT02308072
			I	NCT02229656
	Niraparib	R/M HNSCC	II	NCT04313504
		HNC	II	NCT05169437
			II	NCT04779151
Smoothened	Sonidegib	R/M HNSCC	I	NCT04007744
VEGFR	Bevacizumab	Localized HNSCC	II	NCT01588431
		HNC	III	NCT05063552 NCT00588770
		R/M HNSCC	III	
		Localized HNSCC	I and II	NCT03134846
		HNC	II	NCT03818061
	Lenvatinib	R/M HNSCC	II	NCT04977453
	Cabozantinib	HNC	II	NCT05136196
			I	NCT03170960
		HNSCC	I	NCT03667482
		R/M HNSCC	II	NCT03468218
		R/M HNC	I	NCT04514484
	Sorafenib	R/M HNSCC	II	NCT00494182
	Surufatinib	R/M HNC	II	NCT04910854
	Abemaciclib	R/M HNSCC	II	NCT03356223
		HNSCC	II	NCT04169074
	Ribociclib		I	NCT04000529

HNC—Head and Neck Cancer; HNSCC—Head and Neck Squamous Cell Carcinoma; R/M—Recurrent/metastatic.
